# Can Emotional Competence Be Taught in Higher Education? A Randomized Experimental Study of an Emotional Intelligence Training Program Using a Multimethodological Approach

**DOI:** 10.3389/fpsyg.2018.01039

**Published:** 2018-06-27

**Authors:** Raquel Gilar-Corbí, Teresa Pozo-Rico, Barbara Sánchez, Juan Luis Castejón

**Affiliations:** Developmental and Educational Psychology Department, University of Alicante, Alicante, Spain

**Keywords:** emotional intelligence, multimethodological approach, e-learning methodology, higher education, emotional competence training programs

## Abstract

Since the introduction of the Bologna Process, the goal of education has been not only to acquire technical skills but also to master other skills, such as teamwork, effective communication skills, time optimization, and the ability to manage one's emotions. The present work describes a program to develop emotional intelligence in higher education, the “Emotional Intelligence Training Program,” with a multimethodological approach that offers the opportunity for university students to develop their emotional intelligence. A total of 192 higher education students participated in this educational experience. Of the participants, 66% were women, and 34% were men; the average age of the sample was 18.83 years with a standard deviation of 2.73. The results indicate that our program can help improve emotional intelligence through three proposed methodologies: online, in the classroom, and coaching. It has been demonstrated that the program is effective in the three methodological modalities presented, offering a range of possibilities to future users because it is possible to select the most appropriate modality based on the resources and possibilities available in each situation. Finally, Future research should focus on the application of this program to assess the acquisition of emotional competences at the postgraduate level.

## Introduction

### Theoretical framework

It is important to carry out this study because, although the acquisition of technical and theoretical knowledge is fundamental and necessary in higher education, in our current social reality, it is no longer sufficient (Baker et al., 2013; Rivers and Willans, 2013; Murphy, 2014; Di Fabio and Bucci, 2016). Students must also develop values and attitudes that guide the transfer and applicability of pure knowledge to real challenges and scenarios of personal, social, academic, and professional progress (Griffin and Reason, 2010; Zepke et al., 2014; Theron and Bitzer, 2016).

The types of skills that were promoted decades ago in university education, although they remain necessary, are no longer sufficient (Laux and Franze, 2010; Mocanu et al., 2014; Eurico et al., 2015; Standley, 2015).

For example, aspects such as the promotion of academic intellectual capacities based on inductive and deductive reasoning and on the classical theories of academic intelligence are fundamental to the training of university students; however, in recent decades, the approach to training by competences supports the holistic vision of these training years with the inclusion of social and emotional skills (Silva et al., 2013), critical reasoning, creativity, and the promotion of autonomy and personal initiative for lifelong learning (Pavlin and Svetlik, 2012; Petrovici, 2014; Mok et al., 2016).

The paradigm shift in university education is connected to the discovery of specific talents. In certain educational systems constrained by the exclusive recognition of academic abilities, there is a risk that the potentialities of certain individuals with respect to all of the complexity that characterizes them and the infinite talents and multiple intelligences that define them are not recognized, encouraged, and developed for the benefit of the individual (Rhee and Sigler, 2014; Sartori and Tacconi, 2016).

The current knowledge society offers an extraordinary range of stimuli and options for learning that, at times, overwhelms the individual. One of the fundamental competences is to learn to manage a multitude of sources of information (Sartori and Ceschi, 2013), to know how to discern the vital from the trivial, and to build one's own genuine knowledge, all of which are possible with the same guarantees of success in conventional classroom classes and using e-learning (Liu and Chen, 2013; Lopes et al., 2014; Gerken et al., 2016).

So, the reason it has been decided to verify the effectiveness of methodologies supported in information and communications technologies (ICTs), such as online and coaching used in our study, is because there have been several studies (Deichert et al., 2016; Peterson, 2016) comparing student outcomes from flipped or inverted classrooms to more traditional lecture formats, and the results revealed that students in flipped classrooms produce similar or superior rates of learning retention to those observed in traditional course formats and even beat their classmates in the final exam. Further, these students were more satisfied with the course overall, a novel finding in this burgeoning area of research.

In the same manner, UNESCO considers that ICTs help to promote universal access to education and improve equality and the quality of education. ICTs provide us with the ability to interrelate with students more frequently and allow access to classes using “multimedia applications” for students who cannot access classes in person (Rosario, 2006).

Our universities offer many experiences of “virtual teaching,” “virtual classrooms,” and so on. Institutions must promote innovative experiences in teaching-learning processes, using ICT and relying on changes in teachers' teaching strategies, communication, and distribution systems for learning materials, among other factors (Salinas, 2004; Deichert et al., 2016; Peterson, 2016).

One of the greatest difficulties is to link the learning achieved in the university academic environment with the real challenges present in the workplace and to guide the students toward a productivity of high social value (Guerra and Velasco, 2010; Maher, 2011; Llinares et al., 2013; Thompson et al., 2013; Petrova, 2015; Vande Wiele et al., 2015).

The question that we want to address is whether, as other studies propose (Lindebaum and Cartwright, 2011; Barbieri and Majer, 2012; Lindebaum, 2012), it is essential to master the emotions present in the learning process, understood as a continuum in the internalization of teaching based on a social process of enrichment, communication, and collaboration marked by orientation toward quality and continuous improvement (Miller, 2010; Avsec, 2012; Mikolajczak et al., 2012; Grotkowska et al., 2015) to achieve the objectives previously mentioned. For this reason, we propose our training through the “Emotional Intelligence Training Program” (EITP) in three possible modalities (online, in the classroom, and coaching).

In this way, it is important to say that the acquisition of knowledge is mediated by emotions (Khosla et al., 2009; Miller, 2010; Yip and Côté, 2013). If a positive climate is offered in the university environment for the mastery of optimized emotional management, the experiences of multiple teachings are strengthened and reach completeness in their internalization and implementation by individuals (Woods, 2010, 2012; Erkutlu and Chafra, 2014; Goggin et al., 2015).

Fundamental to this process are those learning experiences that encourage the multiple intelligences and specific talents present in the university student body (Akerjordet and Severinsson, 2008; Gutmann and Interactive, 2008; Neviarouskaya et al., 2008; Zakharov et al., 2008; Jennerich and Oberg, 2009).

This means accepting the positive components of the current university education system and its quality, and improving upon areas based on a conception of academicism (and even industry training) to incorporate the development of key socio-emotional competences. This approach enables students to develop their capacity for improvement, resilience, and coping with adversity and their ability to work under pressure and commits them to thinking about their future outlook and their personal and professional well-being during their development both as individuals and as part of society (Khosla et al., 2009, 2010; Cooper and Ng, 2010; Higgs et al., 2010; Muna and Zennie, 2010; Parker, 2010; Kwon et al., 2013).

A few years ago, university education was based on the notion of a master class with watertight knowledge in specific subjects, rigid schedules that recorded times artificially, groupings by criteria such as chronological age (and not mental maturity), and standardized tests to evaluate performance understood exclusively in terms of the memorization of information. Currently, however, such a paradigm is not sufficient. It is necessary to climb up one more step in the conception of higher education and in the models of learning, considering that ICT can be an equally valid model for the construction of knowledge and for the acquisition of emotional competences (Boza et al., 2014; Gu et al., 2014; Andres and Marieta, 2015; Caballero et al., 2015; Cuenca et al., 2015; Scheepers and Maree, 2015).

## Methods

### Participants

A total of 192 university students participated in this educational experience: 48 students were randomly assigned to the control group and 144 to the experimental group. In the same manner, the 144 individuals of the experimental group were again assigned randomly to the various training modalities, composing a group of 48 students for the classroom modality, another group of 48 students for the online modality, and a final group of 48 students for the coaching modality. Of this total, 66% were women and 34% were men, and the average age of the sample was 18.83 years with a standard deviation of 2.73. Table 1 shows the sociodemographic data of the sample.

**Table 1:** Sociodemographic data of the sample.

	**Group**	**X**	**Standard dev**.	***N***
Age	1	19.27	3.67	48
	2	19.07	2.50	48
	3	18.40	1.10	48
	4	19.54	2.75	48
	**Group**	**% Male**	**% Female**	***N***
Gender	1	25.0	75.0	48
	2	43.8	56.3	48
	3	33.3	66.7	48
	4	47.9	52.1	48

### Instruments

In the present study, two perspectives were combined to evaluate emotional intelligence (EI). On the one hand, EI was measured as a perceived trait for which the Emotional Quotient Inventory: Short (EQ-i:S) was used (Bar-On, 2002). Second, EI was also measured as a skill for which the Situational Test of Emotional Understanding (STEU) and Situational Test of Emotion Management (STEM) were used (MacCann and Roberts, 2008). The following are the instruments selected for the study, the referenced authors, and the primary psychometric qualities. In all cases, participants completed all instruments.

1. The EQ-i:S (Bar-On, 2002) is a short version of the Emotional Quotient Inventory adapted to Spanish by MHS, Toronto, Canada. It comprises 51 items rated on a five-point Likert scale and evaluates five general factors of EI: intrapersonal intelligence (self-perception), interpersonal intelligence, adaptation, stress management, and mood. In the validation sample, the EQ-i:S demonstrated adequate validity and internal consistency of its subscales ranging from 0.65 to 0.86. Examples of EQi items are “It's hard to express my intimate feelings” (item 33) and “I generally expect things will turn out all right, despite setbacks from time to time” (item 42).
2. The STEU and Situational STEM (MacCann and Roberts, 2008) are two skills tests. The first measures the understanding of one's own and other people's emotions, and the second measures emotion management. In both cases, key situations arise-that is, practical cases that are well contextualized so that the participant can indicate, which of the proposed alternatives best responds to the situation (in the first case, by discriminating the key emotion and, in the second, by the selection of the course of action that would be most effective in managing the question posed in an emotionally efficient and intelligent manner). In its original version, the STEU comprised 42 items and STEM, 44; however, in this study, the short versions of 25 and 20 items, respectively, were used. The reliability coefficient in the validation sample was 0.71 for the STEU and 0.92 for the STEM. An example of an STEU item is “An unwanted situation becomes less likely or stops altogether. The person involved is most likely to feel: (a) regret, (b) hope, (c) joy, (d) sadness, (e) relief” (the correct answer is relief). Total scores are calculated by taking the mean of all item scores. An example of a STEM item is “Pete has specific skills that his workmates do not, and he feels that his workload is higher because of it. What action would be the most effective for Pete? (a) Speak to his boss about this, (b) Start looking for a new job, (c) Be very proud of his unique skills, (d) Speak to his workmates about this.”

### Procedure

First, all students were properly informed of the investigation, and their informed consent was requested, detailing the objectives of the study, the responsible team, and the confidentiality of their answers.

Next, the participants were randomly assigned to either the control group or the experimental group. Both the control group and the experimental group completed the pretest questionnaires before the implementation of the program. Then, the experimental groups participated in the program, each in its corresponding training modality. Finally, both groups (experimental and control) completed the posttest questionnaires.

On the other hand, in relation to the intervention itself, we used the EITP, a pedagogical intervention that can be implemented by three different methodological modalities:

1. A classroom-mediated modality combined with an e-learning platform based on a flipped classroom teaching and learning methodology. Individualized and face-to-face tutorials are made to monitor the optimal progress in the acquisition of emotional competences.
2. An exclusively online modality implemented on an e-learning platform and dynamized by forums and activities delivered in a dialogical learning environment. Individualized online tutorials are made to monitor the optimal progress in the acquisition of emotional competences.
3. A coaching-mediated modality combined with an e-learning platform enriched with individualized face-to-face tutorials to monitor the optimal progress in the acquisition of emotional competences.
  The three modalities are taught in seven sessions (one session per week for a total of seven weeks) with the following objectives:
  First session: Introduction
    Second session: Intrapersonal and self-perception (self-esteem, self-realization, and emotional self-awareness)
    Third session: Interpersonal EI (interpersonal relationships, empathy, and social responsibility)
    Fourth session: Adaptability and decision-making (problem solving and impulse control)
    Fifth session: General mood and self-expression (emotional expression, assertiveness, and independence)
    Sixth session: Stress management (flexibility, stress tolerance, and optimism)
    Seventh session: Emotional understanding and emotion management (create a life and career roadmap and develop a commitment to personal and academic growth and development)
    The three modalities have access to the e-learning platform in which six practical activities are delivered:
    1st session: Knowledge and commitment to e-learning work
    2nd session: Exploration of skills and qualities
    3rd session: Innovative ideas and critical-thinking skills
    4th session: Verbal questionnaire and discussion
    5th session: Participation in the forum
    6th session: Joint elaboration of final conclusions

The online modality only offers the activities through the virtual campus. However, the classroom and coaching modalities include a 2-h, in-class session each week that is cross-sectional in content to the proposed activities in the e-learning environment. In this session, the role of the teacher is to masterfully explain the objectives of each task, share examples, and resolve doubts. The materials remain identical—that is, those that are accessible on the e-learning platform.

Finally, the coaching modality, in addition to the e-learning work and the group classroom session described above, includes a weekly individualized session of ~20 min in which each student is guided and oriented toward his or her personal achievement of the objectives proposed in each of the blocks of work.

It should be noted, therefore, that the added value of the coaching methodology is that classroom training is conducted and, moreover, is cross-sectionally mediated on the parallel e-learning platform and enriched with individualized tutorials to monitor optimal progress in the acquisition of emotional competences.

The very existence of the e-learning platform itself already guarantees that the student can address individual questions using the virtual campus and can share with the entire group, through the forums, the questions that he or she considers appropriate. In fact, dialogical learning and debate are stimulated by this virtual campus.

In short, the themes addressed by the three methodologies are identical: the experiences of fluency, multiple intelligences and talents, personal strengths, creativity, understanding and management of emotions, the search for excellence, and, finally, empowerment and the establishment of positive synergies in high-performance environments. However, the methodology used in each theme varies by the terms with which it is described. For more details, please consult the following link: https://web.ua.es/es/ice/moocs-noocs/mooc/inteligencia-emocional-como-competencia-transversal-en-educacion-superior.html

### Experimental design and data analysis

A quasi-experimental design “with control group” was adopted, measuring the emotional competences acquired with the instruments outlined above following the intervention in both groups (control and experimental) and comparing each of the training modalities (experimental, online, and coaching).

To verify the effectiveness of the EI improvement program implemented, the General Linear Model of Repeated Measures was used. The groups of related dependent variables that represented different measures of the same trait were thus analyzed. In addition, this analysis allowed one or several intra-subject factors to be defined. Using this procedure, a multivariate analysis of variance (MANOVA) and a univariate analysis of variance (ANOVA) of repeated measures were performed in which the measures of the dependent variables were treated as measured variables within the same subjects and the groups acted as variables between subjects. All the statistical analyses were conducted using SPSS V.21.0 (IBM, Armonk, USA).

## Results

The result of the M-Box test indicates the homogeneity of the variance-covariance matrices for the adaptability factor (*F* = 0.665, *p* = 0.742). However, the result does not indicate such homogeneity for the intrapersonal factor (*F* = 4.271, *p* = 0.001), the interpersonal factor (*F* = 2.064, *p* = 0.29), the stress management factor (*F* = 2.994, *p* = 0.001), general mood (*F* = 3.932, *p* = 0.001), the EQ-i Total factor (*F* = 18.837; *p* = 0.001), or the STEU (*F* = 48.303; *p* = 0.001) and STEM (*F* = 71.121; *p* = 0.001). In any case, it should be remembered that a violation of this assumption has a minimum effect if the groups are approximately equal in size (Hair et al., 1999).

Below are the specific results of each of the factors related to EI that were targeted for improvement by the implemented program.

As seen in Table 2 of intra-subject and inter-subject effects, the resulting values of the test indicate that the effect of the interaction between the time of evaluation (pretest and posttest) and the implementation of the program is significant (*p* ≤ 0.05) among experimental Groups 1 (classroom methodology), 2 (online methodology) and, 3 (coaching methodology) compared with Group 4 (control). Therefore, it is evident that the level of competence of the participants in the experimental group indicates significant improvements after the intervention, in the scores of both all of the factors of the EQ-i test and the STEM/STEU measures.

**Table 2:** Summary of intra-inter subject univariate ANOVA.

	**Source**	**Type III**	**df**	**F**	**Sig**.	**η2 partial**	**Ob. power**	***Post-hoc***
Intrapersonal	Intra	1971.094	1	129.600	0.001	0.408	1.001	1 = 2; 2 = 3;
	Intra^*^Entre	561.615	3	12.309	0.001	0.164	1.001	1 < 3; 1>4;
	Error intra	2859.292	188					2>4; 3>4
	Inter	492350.260	1	27732.849	0.001	0.993	1.001	
	Error inter	3337.625	188					
Interpersonal	Intra	978.565	1	117.596	0.001	0.385	1.001	1 = 2; 3>1;
	Intra^*^Entre	299.508	3	11.997	0.001	0.161	1.001	3>2; 3>4;
	Error Intra	1564.427	188					1>4; 2>4
	Inter	686732.086	1	71354.950	0.001	0.997	1.001	
	Error inter	1809.344	188					
Stress management	Intra	1056.690	1	68.816	0.001	0.268	1.001	1 = 2; 3>1;
	Intra^*^Entre	1235.008	3	26.810	0.001	0.300	1.001	3>2; 3>4;
	Error Intra	2886.802	188					1>4; 2>4
	Inter	304707.003	1	14901.737	0.001	0.988	1.001	
	Error inter	3844.177	188					
Adaptability	Intra	997.815	1	128.577	0.001	0.406	1.001	2 = 3; 2>1;
	Intra^*^Entre	372.716	3	16.009	0.001	0.203	1.001	3>1; 1>4;
	Error Intra	1458.969	188					2>4; 3>4
	Inter	273013.336	1	25798.562	0.001	0.993	1.001	
	Error inter	1989.510	188					
General mood scale	Intra	7938.844	1	734.416	0.001	0.796	1.001	3>1; 3>2;
	Intra^*^Entre	959.927	3	29.601	0.001	0.321	1.001	3>4; 2>1;
	Error Intra	2032.229	188					2>4; 1>4
	Inter	629208.167	1	38671.715	0.001	0.995	1.001	
	Error inter	3058.854	188					
Total EQi	Intra	2095.336	1	1793.872	0.001	0.905	1.001	3>1; 3>2;
	Intra^*^Entre	583.570	3	166.537	0.001	0.727	1.001	3>4; 2>1
	Error Intra	219.594	188					2>4; 1>4
	Inter	461884.888	1	111557.977	0.001	0.998	1.001	
	Error inter	778.379	188					
STEU	Intra	1236.253	1	1187.877	0.001	0.863	1.001	2 = 3; 2>1;
	Intra^*^Entre	508.591	3	162.896	0.001	0.722	1.001	3>1; 1>4;
	Error Intra	195.656	188					2>4; 3>4
	Inter	70986.565	1	14678.967	0.001	0.987	1.001	
	Error inter	909.156	188					
STEM	Intra	517.072	1	882.645	0.001	0.824	1.001	1 = 2; 3>1
	Intra^*^Entre	209.922	3	119.446	0.001	0.656	1.001	3>2; 3>4
	Error Intra	110.134	188					1>4; 2>4
	Inter	42746.786	1	18066.174	0.001	0.990	1.001	
	Error inter	444.831	188					

Within the experimental group, significant differences were also observed among the three methodologies used; however, it was the coaching methodology (Group 3) that achieved the best results in teaching EI. Table 3 presents the pretest and posttest means of each of the groups.

**Table 3:** Marginal means.

	**Group**	**X Pre-test**	**Standard dev. Pretest**	**X Post-test**	**Standard dev. post-test**	**N**
Intrapersonal	1	32.7292	2.98749	37.8750	3.71670	48
	2	32.6250	6.13197	40.1875	3.26592	48
	3	35.7708	3.91527	40.3750	4.60631	48
	4	33.0417	3.87550	33.8542	3.04568	48
Interpersonal	1	40.3125	3.34636	44.4583	2.40530	48
	2	39.9583	3.77539	44.4167	3.14090	48
	3	42.0625	2.46167	46.0833	2.59158	48
	4	40.4375	3.45122	40.5833	2.44804	48
Stress management	1	26.9792	3.59367	29.3125	3.80457	48
	2	23.5833	5.16054	32.3958	3.23373	48
	3	29.5833	3.83618	32.8958	3.72021	48
	4	25.8958	4.38683	24.7083	5.56187	48
Adaptability	1	24.1667	2.87567	28.1042	2.93359	48
	2	24.9583	3.55479	30.4792	2.68954	48
	3	27.0208	2.97142	30.3542	2.99993	48
	4	24.0625	3.04859	24.1667	3.08278	48
General mood scale	1	34.1875	4.02469	45.6875	2.16506	48
	2	36.2917	4.69476	47.3750	3.17319	48
	3	38.4167	3.03759	48.5208	3.55498	48
	4	34.8333	3.74355	38.5208	4.40980	48
Total EQi	1	31.6750	1.48159	37.0875	.71090	48
	2	31.4833	2.59421	38.9708	1.14779	48
	3	34.5708	1.06949	39.6458	2.30161	48
	4	31.6542	1.44737	32.3667	1.39625	48
STEU	1	10.4167	1.55513	15.0208	.14434	48
	2	11.9375	2.98511	18.2708	.89299	48
	3	14.3958	1.60769	17.7708	2.03417	48
	4	10.4583	1.54312	10.5000	1.50177	48
STEM	1	8.6691	1.75565	12.2578	.12596	48
	2	9.0370	1.65141	12.7040	.37070	48
	3	11.1034	.58034	13.0945	.51640	48
	4	8.7521	1.64735	8.7885	1.58991	48

## Discussion

In recent decades, we have experienced a process of transformation in several social spheres. With the development and use of new technological devices and the Internet (Torkzadeh and Van Dyke, 2002; Weatherbee, 2010) together with the economic crisis (Utting et al., 2012) and the different types of social interaction (Tyler, 2002), modern society has been defined as an information society (Webster, 2002), a knowledge society (Hargreaves, 2003), and even a liquid society (Bauman, 2000, 2011). The pace and expansion of markets, globalization, the effect of new technologies, and high-performance competition scenarios are key developments in today's society (Moss and Richter, 2011; Pavlin and Svetlicic, 2012; Minten and Forsyth, 2014; Casano, 2015; Kalfa and Taksa, 2015).

The new demands and requirements that occur when university graduates leave the classroom no longer refer exclusively to the technical competences ascribed to their university degrees (Pop and Mihaila, 2008; Tomlinson, 2008; Turner, 2014; Mazalin and Kovacic, 2015). This type of theoretical knowledge is necessary but no longer sufficient. Practical skills are also required that allow what has been studied theoretically to be put into practice in real-life scenarios. In addition, highly defined attitudes of motivation, self-determination, overcoming, enthusiasm, and the ability to deliver are also necessary. Similarly, skills that enable teamwork, cooperation, and the assumption of leadership and subordination roles are required depending on the moment and need and, occasionally, are even required simultaneously (Ceschi et al., 2014). As in other studies (Cunico et al., 2012), our program demonstrated that emotional competence can be taught and effectively learned by students, constituting a commitment to teach not only theoretical knowledge but also skills—in this case, of an emotional nature—that students can use to achieve their personal, academic, and professional goals.

For all of the above, the educational systems and, especially, the universities play an important role in making decisions regarding the training and education of their students with the goal of offering training that covers the possible challenges that the students must address, particularly at the beginning of their professional careers but also throughout their careers (Maher, 2011; Extremera et al., 2012; Silva et al., 2013).

The results obtained in the present work demonstrate the effectiveness of our program for EI development across any of the three proposed modalities (online, in the classroom, and coaching) with the greatest impact on coaching. EI is key to overcoming the challenges that university students will face when they leave the classroom, during their careers, and in their personal and professional futures. Other research is in line with these results we have obtained in higher education (Oriol et al., 2016; Torrecilla-Sánchez et al., 2018). In fact, EI is key to overcoming the challenges that university students will face in these periods (Pavlin and Svetlik, 2012; Mok et al., 2016).

In addition, various investigations have worked in the same line on the successful implementation of IE training programs since childhood (Denham et al., 2012; Fernández-Sánchez et al., 2015), adolescence (López-González and Oriol, 2016), and adulthood within the university education, both undergraduate (Oriol et al., 2016) and master's-level (Torrecilla-Sánchez et al., 2018).

In the same line, in investigations such as Oriol et al. (2016), it is concluded that university students can understand the complexity of the emotional processes they undergo. A greater control of these emotions would allow students to maintain higher levels of interest in their studies at the different educational stages and to avoid the risk of school failure.

Consistent with this perspective, our goal is to teach skills that provide access to a full life, even beyond the professional sphere (Kruss, 2004; Guerra and Velasco, 2010; Petrova, 2015; Vande Wiele et al., 2015). EI skills provide the individual with a comprehensive education for life that will help in overcoming key obstacles, addressing problems, and maintaining perseverance in achieving one's own objectives, beliefs, and values in a flexible but continuous manner, just as EI is achieved by the program presented here.

In short, consistent with what has been discussed, our program enables teaching EI in the three modalities presented: classroom, online, and coaching. All of the modalities are effective, allowing the most appropriate method to be determined according to the resources and possibilities that are available at the time. This training approach encourages a continuous commitment to improving curricula and providing more comprehensive training with which students can address future challenges.

Finally, it should be noted that there are three main limitations of this study. First, the sample consists of groups of 48 students; it would be interesting to have a larger sample of students to see if the results can be generalized. In the same way, it would be beneficial to include a follow-up of the students to know if the acquired emotional competence is maintained over time and is resistant to the challenges and new situations that the students must face in their personal and academic lives. Finally, and in line with other research carried out with the aim of training in EI, it would be very convenient to know if the acquisition of emotional competence has a positive impact on the academic performance of the students. All these issues are considered as future lines of intervention.

## Conclusions

Our study demonstrated that emotional competence can be taught in higher education and that the university environment presents the ideal climate in which to optimize the emotional management that strengthens multiple learning experiences. Thus, the student will be able to fully achieve the internalization and implementation of these teachings. This emotional domain will undoubtedly enable these individuals to face challenges not only in their current and future professional arenas but also in other spheres of their lives, helping them achieve well-being and personal happiness. This will occur by strengthening their tenacity, ability to deliver, perseverance, and determination to successfully address key moments such as graduating, entering the workforce, and inclusion as active and productive members of society (Heck and Johnsrud, 1994; Gibbs, 2010; Woods, 2012; Mocanu and Zamfir, 2014; Poortman et al., 2014).

Several authors have proposed that the acquisition of knowledge is mediated by emotions (Khosla et al., 2009; Miller, 2010; Opengart and Bierema, 2015). If a positive climate is offered in the university environment for the mastery of optimized emotional management, the experiences of multiple learning are strengthened and reach completeness in their internalization and implementation. The individuals who succeed in reaching this completeness will, without doubt, achieve tenacity, the ability to deliver, perseverance, and determination to overcome challenges during key moments such as graduating, entering the workforce, and inclusion as active and productive members of society. These skills will translate into other spheres of their lives and to future challenges throughout their professional careers and their search for well-being and personal happiness (Heck and Johnsrud, 1994; Gibbs, 2010; Woods, 2012; Mocanu and Zamfir, 2014; Poortman et al., 2014; Caballero et al., 2015).

Various investigations indicate that, in the tewny-first century, universities are committed to creativity and the use of ICTs (Casarotti et al., 2002). Universities are committed to the concept of knowledge acquisition linked to the generation of original high-value ideas, derived from an approach that encourages the discovery of a multitude of equally valid manners and paths for solving a problem or achieving a particular goal (Laux and Franze, 2010; Mocanu et al., 2014; Eurico et al., 2015; Sanchez-Hernandez et al., 2015).

In a highly technological world in which increasingly more productive, labor, and social processes are imbued with ICTs, not using these tools to also develop our emotional competence would be like closing our eyes to the reality and environment into which we are inserted and dooming our students to the same darkness. We must not miss the opportunity to offer more real and pragmatic learning environments. ICT tools therefore provide us, visible in the three training modalities of our program, with the instrument to convey the connection of students to the environment and to the elements of the university sphere and various areas of knowledge. These tools also offer us the opportunity to teach and improve the emotional capacity of the agents involved, thus achieving the holistic learning that is currently demanded in our society. Therefore, it is essential to move forward with this program with its three forms of training, by students and for students. Only in this manner will a connection with the environment or appropriate element of the university sphere be achieved for each area of knowledge in a process driven by the empowered emotions, creative processes, and holistic learning conveyed by ICTs (Liu and Chen, 2013; Lopes et al., 2014; Gerken et al., 2016; Torrecilla-Sánchez et al., 2018).

University studies are an ideal environment for empowerment and improvement. Training that is focused on the development of the skills of each university degree is an essential goal of each field of study; however, such training is no longer sufficient (Sokolov, 1977; Trehub, 1977; Brooks, 1989; Gorkin and Shevchenko, 1997; Krause et al., 2006; Ren et al., 2016). In addition, training focused on key emotional competences is required. Consistent with this commitment, our research demonstrates a significant improvement in the level of emotional competence using all three modalities after completing the EITP. Thus, improvement in emotional intelligence is possible through our program in its three variants. However, although both classroom and online methodologies are effective, the highest performance was demonstrated by the coaching methodology. In the coaching methodology, classroom training is conducted and is also cross-sectionally mediated in a parallel e-learning platform and enriched with individualized tutorials to monitor the optimal progression in the acquisition of emotional skills. This process causes us to reflect on the importance of the two personal intelligences in teaching. On the one hand, it is necessary to enhance the capabilities of interpersonal intelligence that enable cooperative and group work as well as leadership capacity. On the other, it is important to maintain a personal and unique relation with each of the participants, a relation that helps the individual to enhance the qualities of intrapersonal intelligence, to be valued as an individual, and to recognize his or her own strengths.

In this manner, the present research seeks to exemplify training at this level of commitment in the curricula of contemporary universities. This training provides a type of education that contemplates the comprehensive education of the individual in addition to academic training. The training also stimulates the teacher's reflection in the sense that what excites us is better able to capture our attention, our curiosity, and our ability to transform, something that undoubtedly translates into a more holistic type of learning.

Finally, this work opens the doors to future research to consider the follow-up of achievements in the acquisition of socio-emotional competences in the postgraduate phase.

## Ethics statement

All participants were properly informed of the investigation, and their informed consent was obtained. All methods were performed in accordance with the relevant guidelines and regulations and the study was approved by University of Alicante Ethics Committee (UA-2015-07-06) and carried out in accordance with the relevant guidelines and regulations.

## Author contributions

RG-C was director of the research and carried out the quantitative methods. TP-R did fieldwork and program implementation. JC worked on quantitative methods. BS carried out a theoretical review of the topic.

### Conflict of interest statement

The authors declare that the research was conducted in the absence of any commercial or financial relationships that could be construed as a potential conflict of interest.
